# Reflective Practice About Retroperitoneal Laparoscopy in Comparison to Open Surgery for Ureteropelvic Junction Obstruction Repair in Children Less Than 1 Year of Age

**DOI:** 10.3389/fped.2019.00194

**Published:** 2019-05-24

**Authors:** Anthony Kallas-Chemaly, Matthieu Peycelon, Liza Ali, Christine Grapin-Dagorno, Elisabeth Carricaburu, Pascale Philippe-Chomette, Goharig Enezian, Annabel Paye-Jaouen, Alaa El-Ghoneimi

**Affiliations:** ^1^Robert-Debré University Hospital, AP-HP; Université Paris Diderot, Sorbonne Paris Cité, Pediatric Surgery and Urology, National Reference Center of Rare Urinary Tract Malformations (MARVU), Paris, France; ^2^Department of Pediatric Surgery and Urology, Faculty of Medicine, Université Saint-Joseph, Hôtel-Dieu de France, Beirut, Lebanon

**Keywords:** ureteropelvic junction obstruction, open surgery, retroperitoneal laparoscopy, feasibility, benefits

## Abstract

**Introduction:** The interest in laparoscopy in the treatment of ureteropelvic junction obstruction (UPJO) in children under 12 months of age remains controversial. The aim of this study is to evaluate feasibility and benefits of retroperitoneal laparoscopy (RL) compared to open surgery in this age group.

**Materials and Methods:** Between January 2012 and May 2017, we performed 222 pyeloplasties: 144 by laparoscopy and 78 by open surgery. From 2012, the choice of operative technique was decided according to the laparoscopic experience of the surgeon; two surgeons operated laparoscopically on all children <12 months of age, while others operated using posterior lumbotomy (PL). The RL is standardized and performed by 3 trocars (5, 3, 3). Pre, per and postoperative parameters were analyzed retrospectively. Statistical tests: Pearson, Fisher, Student and Mann-Whitney.

**Results:** During this 5-year period, 24 RL and 53 PL were included with a median follow-up of 27 months (5–63). In the LR group, postoperative drainage was performed by JJ (13 cases) and external stent (11 cases). No conversion has been listed in this group. In each group there was one failure that needed redo pyeloplasty. Duration of hospitalization and intravenous acetaminophen use were significantly lower in the RL group (2.8 vs. 2.3 days, *p* = 0.02, respectively) while operating time was significantly longer (163 vs. 85.8 min, *p* = 0.001). The postoperative complication rate was statistically identical in each group (urinary tract infection, wall hematoma, hematuria…).

**Conclusion:** RL is feasible in children under 1 year of age in the hands of well-experienced surgeons with longer operative time but without added morbidity. Subject to the retrospective nature of our study, the RL seems to offer a benefit regarding duration of hospitalization and analgesics consumption.

## Introduction

**Figure 1:**
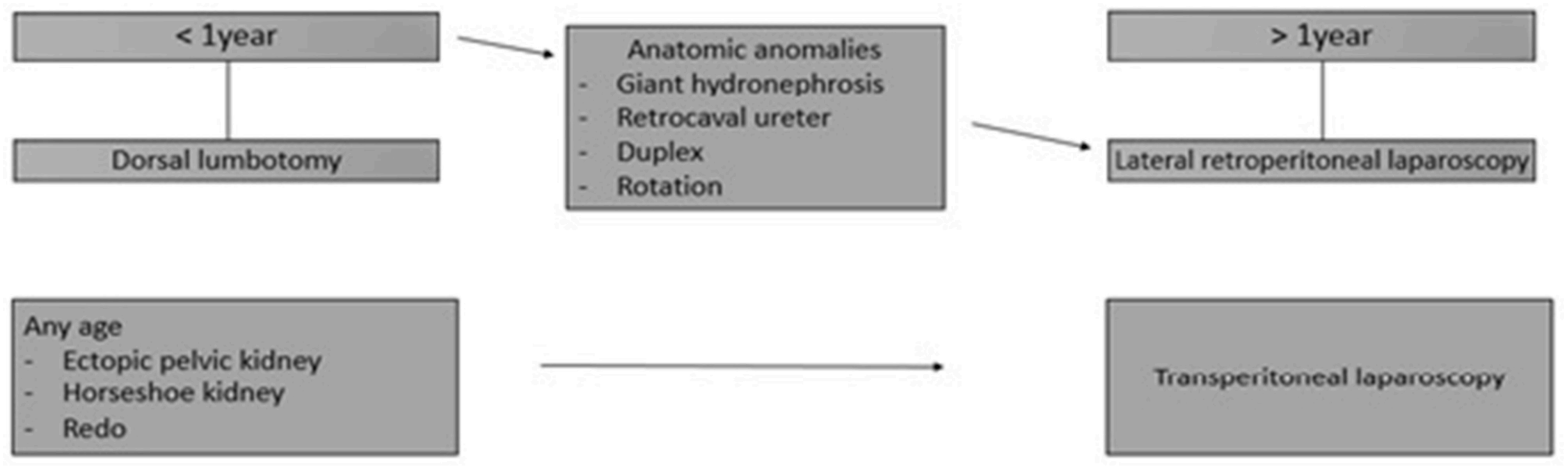
Our strategy for UPJO management before 2012.

Meta-analyses, retrospective and prospective studies have shown over time the explicit benefits of laparoscopic treatment of the ureteropelvic junction obstruction (UPJO) compared to open surgery (1, 2). In addition, retroperitoneal laparoscopic pyeloplasty (RL) appears to offer more advantages compared to the transperitoneal laparoscopic (TL) approach (3). Although some surgeons have adopted the transperitoneal route for laparoscopic pyeloplasty in infants (4–6), RL remains controversial in children under 1 year of age and is not considered in many pediatric urology centers because of technical difficulties encountered and lack of proven benefits. Before 2012, our strategy for UPJO management was essentially to operate children under 1 year of age by posterior lumbotomy (PL) and those over 1 year by RL (Figure 1). After 2012, following extensive experience in laparoscopic surgery, two pediatric urologists decided to start RL in children under 1 year of age.

The objective of our retrospective study was to evaluate the feasibility and benefits of RL in this age group in order to standardize management in our department. We found it necessary to compare the results of RL to those of open surgery. To our knowledge, this is the first study comparing the two approaches exclusively in children under 12 months of age.

## Materials and Methods

Between January 2012 and May 2017, we performed 222 pyeloplasties: 144 by laparoscopy and 78 by open surgery. 82 were first cases of children <1 year of age: 55 operated by PL and 27 by laparoscopy (24 by RL and 3 by TL). The 3 TL cases were related to a child with an ectopic kidney and two children with a horseshoe kidney.

The 55 PL and 24 RL cases were diagnosed antenatally. Indications for surgery were a progressive increase in antero-posterior pelvis diameter (APPD), APPD >30 mm, relative renal function of <40% with Uro-MRI or with MAG-3 renal scintigraphy, and febrile urinary tract infection. Preoperative evaluation included renal and bladder ultrasound, Uro-MRI, or MAG-3 renal scintigraphy, and retrograde cystography in case of febrile urinary tract infection.

The RL is standardized in our department as previously described by Blanc et al. (7). We use a 5 mm optic trocar and two 3 mm working trocars. The ureteropelvic anastomosis is performed using the Hynes-Anderson technique with a resorbable monofilament 6–0 (3/8 circle needle) and with a JJ or external transpelvic stent (Supplementary Video 1). We do not use a perirenal drain. The bladder catheter is left for 24 h. Antibiotic prophylaxis is done preoperatively (a dose of ceftriaxone of 50 mg/kg). The JJ probe is removed at ~6 weeks under general anesthesia and the external stent is clamped on day 1 postoperatively and removed in consultation at 10 days from the operation.

The PL is performed according to the same principles by an extraperitoneal muscle splitting posterior approach using a transverse cutaneous incision at mid-distance between the last rib and the iliac crest.

Mean postoperative follow-up was 27 months (range 5–63) and consisted of a clinical visit associated with renal and bladder ultrasound at 1 month after stent removal, and then every 3 months for the first year. In case of favorable evolution (absence of symptoms and significant decrease in dilatation), follow-up was done annually for 5 years. A functional renal evaluation (MAG-3 or uro-MRI) is not performed systematically but in case of significant asymmetry of preoperative function and/or clinical or radiological abnormalities postoperatively. Almodhen et al. (8) have already shown the reliability of this protocol. Success was considered objectively as a resolution or a reduction of hydronephrosis on renal ultrasound (APPD).

We compared the preoperative (age, sex, weight, laterality of the UPJO and the APPD on renal ultrasound), intraoperative (operative duration, type of pelvic drainage, operator, conversion) and postoperative (operative time, duration of hospitalization, duration of acetaminophen IV administration, nalbuphine IV administration, APPD on renal ultrasound after more than 3 months from the operation, and complications in the short and long-term) criteria between the two groups.

Two bilateral cases in the PL group were excluded from the study given the absence of bilateral cases in the RL group. Six cases in the PL group associated with simultaneous interventions that may affect operative time, pain analysis and hospital stay (1 case of gastrostomy, 2 cases of inguinal hernia repair and 3 cases of circumcision) have been excluded from these parameters analysis.

The statistical analysis was done by SPSS (Statistical Package for Social Sciences), version 13.0. A difference is noted as significant if *p* < 0.05. Categorical variables were compared according to the Pearson *X*^2^ test or the Fischer F test, and quantitative variables according to the Student *t*-test or the Mann-Whitney *U*-test.

## Results

The two groups, PL and RL, were homogeneous according to age, sex, weight, laterality of the UPJO, and preoperative APPD on renal ultrasound. Patient demographics are summarized in Table 1.

**Table 1:** Patients demographics.

	**PL (*n* = 53)**	**RL (*n* = 24)**	***P*-value**
Age (month) (SD)	5.2 (2.6)	7.1 (3.87)	0.06 (U)
Sex, *n* (%)			0.90 (*X*^2^)
Boys	41 (77.3)	19 (79)	
Girls	12 (22.7)	5 (21)	
Weight (kg) (SD)	7.67 (1.3)	7.97 (1.7)	0.14 (*t*)
Laterality of UPJO, *n* (%)			0.30 (*X*^2^)
Right	22 (41.5)	11 (45.8)	
Left	31 (39.5)	13 (54.2)	
Pelvis diameter (mm) (SD)	28.9 (9.7)	26.9 (11.7)	0.43 (*t*)

*SD, Standard Deviation; n, number of patient*.

Duration of operation was greater in the RL group (163 vs. 85.8, *p* = 0.001). Pelvis drainage was performed by an external stent in 94.3% of PL cases and 46% of RL cases (*p* = 0.001). Fellows were first operator in 34% of PL cases and in 12.5% of RL cases (*p* = 0.01). No cases of aberrant polar vessels were noted in either group. No conversion cases were recorded in the RL group. Intraoperative data are summarized in Table 2.

**Table 2:** Intraoperative data.

	**PL (*n* = 53)**	**RL (*n* = 24)**	***P*-value**
Median operative time (minutes) (SD)	*n* = 47	*n* = 24	
	85.8 (20.4)	163.0 (36.4)	0.001 (*t*)
Stent, *n* (%)	*n* = 53	*n* = 24	0.001 (*X*^2^)
Double J	3 (5.7)	13 (54)	
External stent	50 (94.3)	11 (46)	
Polar vessels, *n* (%)	*n* = 53	*n* = 24	
Aberrant	0	0	
Non-aberrant	0	3 (12.5)	
Operator, *n* (%)	*n* = 53	*n* = 24	
Senior	35 (66)	21 (87.5)	0.001 (*X*^2^)
Fellow	18 (34)	3 (12.5)	

A significant difference in favor of RL was demonstrated regarding the duration of hospitalization and duration of intravenous acetaminophen administration. On the other hand, this difference was not significant with regard to the duration of nalbuphine intravenous administration. APPD on renal ultrasound at more than 3 months from the operation was identical in both groups. These postoperative parameters are summarized in Table 3.

**Table 3:** Postoperative parameters.

	**PL**	**RL (*n* = 24)**	***P*-value**
Length of hospital stay (days) (SD)	*n* = 47		
	2.8 (0.9)	2.3 (0.6)	0.02 (*U*)
Postoperative IV acetaminophen use (days) (SD)	*n* = 47		
	2.8 (0.9)	2.3 (0.6)	0.02 (*U*)
Postoperative IV nalbuphine use (days) (SD)	*n* = 47		
	1.9 (0.9)	1.8 (0.5)	0.83 (*U*)
Postoperative US pelvic diameter (mm) (SD)	*n* = 53		
	12.3 (8.6)	12.4 (7.9)	0.85 (*U*)

All postoperative complications were grade I or II according to the Clavien-Dindo classification (9), with the exception of one case in each group (grade IIIb). In fact, all early complications resolved spontaneously or by medical treatment, without the need for surgical management. Only one patient in the PL group and one in the RL group were surgically reoperated by LT for UPJ stenosis at 1 year and 4 months respectively from the first operation. We had no case of urinary fistula, urinoma, or malposition of the stent. Complication rates were identical in both groups. The complications encountered are summarized in Table 4.

**Table 4:** Postoperative complications.

	**PL**	**RL**	***P*-value**
Hematuria, *n*	1	1	0.8 (*F*)
Hematoma, *n*	1	0	0.9 (*F*)
Wound dehiscence, *n*	2	0	0.6 (*F*)
Urinary tract infection, *n*	7	3	0.2 (*X*^2^)
Recurrent UPJO, *n*	1	1	0.9 (*F*)

## Discussion

Laparoscopic pyeloplasty remains a fundamental element of discussion in pediatric urology. A clear advantage of laparoscopy over open surgery has been proven by both retrospective and prospective trials with the same success rate (1, 2). RL for UPJO was reported for the first time in 2001 by Yeung et al. in 13 patients (10). Another preliminary experiment in 22 children was reported in 2003 by El-Ghoneimi et al. (11). In this series, four children required conversion to open surgery. We have already compared in a retrospective study the results of RL (*n* = 22) compared to open pyeloplasty by lumbotomy (*n* = 17) in children over 2 years of age (12). Although the operating time of the RL was significantly longer, the main advantage was a reduced stay in the hospital. The use of analgesics was also reduced after laparoscopy.

There are still controversies about retroperitoneal laparoscopic pyeloplasty in small children. The technical difficulty of this approach probably explains why it remains unavailable even in certain major centers. However, we believe that the exposition of the renal pelvis, the ureter and, occasionally, the polar vessels was excellent, and the working space for suturing and knotting was adequate, including in children younger than 1 year as suggested in work by Metzelder (5). Valla el al. reported feasibility of the retroperitoneal laparoscopy in 8 children <12 months of age. No case of conversion was reported. No recurrence was noted in this small group and early complication rate was similar to the open group (13).

We systematically perform all laparoscopic renal surgery by RL in our department, and we reserve the transperitoneal approach for selected indications. We believe that in the experience of pediatric urology specialist departments, the team should be familiar with both approaches and should decide what is the easiest for the team to apply regularly, keeping the alternative approach for particular indications. The choice to perform TL or RL approach should be based primarily on personal preferences and experience of the individual surgeon.

The current technique in our department was started in 1999 by El-Ghoneimi and has been modified several times since. We first used a resorbable monofilament 5.0 for the ureteropelvic anastomosis and then with the development of 3 mm instruments, we moved to 6.0, thus remaining confident of reproducing the same principles of open pyeloplasty. Since then, we have no longer used a suction drain around the anastomosis. Another change was to limit the number of trocar to 3 instead of 4, allowing to work more freely in an already limited space. Pelvic drainage represents an important point of evolution in our experience; the use of the external stent makes it possible to avoid the general anesthesia necessary for removal of the JJ probe (14).

It was after 13 years of experience that the idea of operating the UPJO by RL in children under 1 year was given due consideration in our department. Laparoscopic pyeloplasty in patients younger than 12 months is rarely considered because of potential problems with hemodynamic and respiratory disorders, the difficulty of the technique, and most importantly, the rapid recovery from open surgery in patients within this age group (15, 16). The risk of conversion is a major element; Badawy et al. reported in a prospective study that the risk of conversion after RL is seen mainly in children under 3 months of age (17). In our series, no case of conversion has been reported; the smallest child of the RL group was of 6 weeks of age with a weight of 4.8 kg. The placement of the external stent in RL cannot be done via the parenchyma but via the pelvis with a theoretical risk of leakage through the drainage point. However, we had no complications of urinoma or malposition of the external stent. Our trend now is to place an external stent whenever the anatomical situation would allow it.

To our knowledge, our series is the first in the literature that exclusively compares PL and RL in the treatment of UPJO in children under 12 months of age. The success rate and complications are identical in both groups. With RL, we obtained a shorter hospitalization stay and less postoperative pain in spite of a longer operating time. Although our current study is retrospective, the same pain management protocol was followed in all children and the same pain score treatment was strictly respected in the department. However, a prospective randomized study is required to confirm this result. From a cosmetic point of view, it seems to us that the scars created by 3 mm trocars are better than the scars of open surgery.

One of our main concerns is teaching RL to fellows or to qualified surgeons with recent experience in advanced laparoscopic surgery. A training program for RL was set up in 2010 (7) and can be actually applied for fellows concerning retroperitoneal laparoscopy in children under one year of age. In our series, 12.5% of RL cases were operated by a surgeon in training. Minimal invasive pyeloplasty in infants of <1 year of age is still limited to few centers. The development of robotic assisted pyeloplasty did not significantly increase the number of minimally invasive pyeloplasties in this group of children (18).

## Data Availability

The datasets for this manuscript are not publicly available because they are related to patients files that are confidential and are found in the archive of Robert-Debré University Hospital. Requests to access the datasets should be directed to alaa.elghoneimi@aphp.fr.

## Ethics Statement

This retrospective study on patients files is not in need for ethical approval or patient's consents according to the French regulation for clinical research. The study was conducted in accordance with the French Legislation, the Good Clinical Practice and the Declaration of Helsinki.

## Author Contributions

As member of the ESPES (European Society for Pediatric Endoscopic Surgeons), I would like to thank the ESPES for their help in writing this manuscript. AK-C and AE-G: study concept and design, acquisition of data, and drafting of the manuscript. AK-C, MP, LA, CG-D, EC, PP-C, GE, AP-J, and AE-G: analysis and interpretation of data. MP, LA, AP-J, EC, GE, PP-C, and CG-D: critical revision of the manuscript for important intellectual content. AK-C and MP: statistical analysis. AK-C, MP, and AE-G: administrative, technical, or material support. AE-G: supervision. All authors contributed for this manuscript.

### Conflict of Interest Statement

The authors declare that the research was conducted in the absence of any commercial or financial relationships that could be construed as a potential conflict of interest.
