# Supplementary figures and images for: Reflective Practice About Retroperitoneal Laparoscopy in Comparison to Open Surgery for Ureteropelvic Junction Obstruction Repair in Children Less Than 1 Year of Age
Source: Front Pediatr. 2019 May 24;7:194. doi: 10.3389/fped.2019.00194 (PMC6543804; doi:10.3389/fped.2019.00194)

## Slide 1
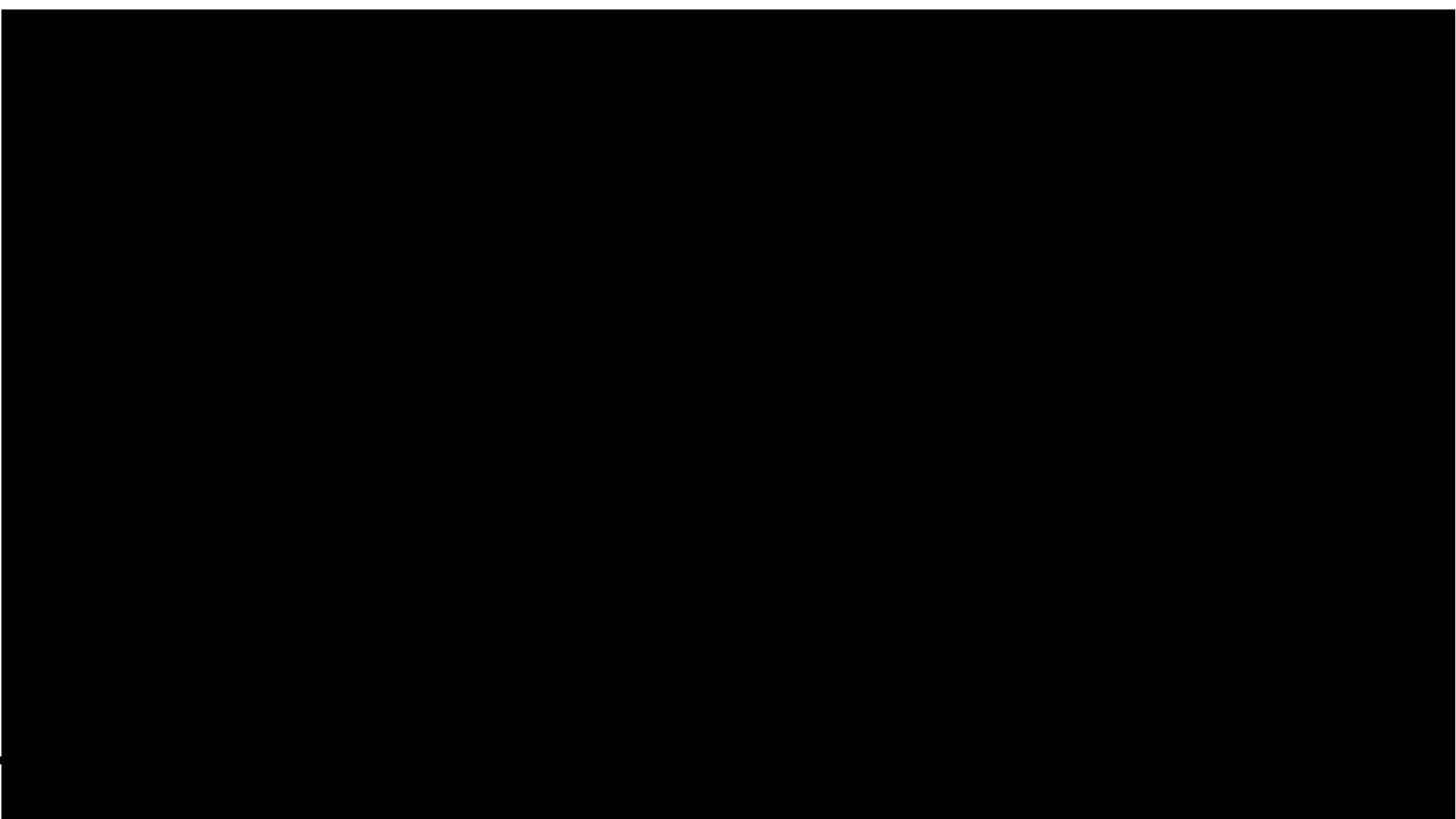

#

Supplement: Supplementary file 1 [file Presentation_1.PPTX]
